# Perioperative Ketorolac and Hematoma Following Breast Reduction: A Systematic Review and Meta-analysis

**DOI:** 10.1007/s00266-025-05187-y

**Published:** 2025-09-19

**Authors:** Victor F. A. Almeida, Glaudir Donato, Manoela Dantas, Eliana F. R. Duraes

**Affiliations:** 1 Department of Anesthesiology, Cleveland Clinic Foundation, Cleveland, Ohio 44195 USA; 2 Center of Medical Sciences, Federal University of Paraíba, João Pessoa, Brazil; 3 Center for Medical Sciences, University Center of João Pessoa - UNIPÊ, João Pessoa, Brazil; 4 Department of Plastic Surgery, Cleveland Clinic Foundation, 9500 Euclid Ave, Cleveland, Ohio 44195 USA

**Keywords:** Ketorolac, NSAIDs, Hematoma, Breast reduction, Reduction mammaplasty, Postoperative bleeding, Analgesia

## Abstract

**Background:**

Ketorolac, a nonsteroidal anti-inflammatory drug, is a promising opioid-sparing option for postoperative pain control. However, its impact on platelet function raises concerns about bleeding risk. Reduction mammaplasty carries a known risk of hematoma, and this study aimed to assess whether perioperative ketorolac increases this risk.

**Methods:**

We searched Cochrane Central, Embase, PubMed, and Web of Science databases for studies involving breast reduction patients who did or did not receive perioperative ketorolac. The primary outcome was hematoma formation, with secondary outcomes distinguishing between cases requiring surgery and those managed conservatively. A random-effects model calculated pooled odds ratios (OR) with 95% confidence intervals (CI), and heterogeneity was assessed using the I^2^ statistic.

**Results:**

Seven studies involving 3,418 patients were included—1991 in the ketorolac group and 1427 in the control group. The hematoma incidence was 6.13% in the ketorolac group versus 6.73% in controls. Meta-analysis revealed a significantly increased risk of hematoma in ketorolac users (OR 2.63, 95% CI 1.58–4.37, *p* < 0.001, I^2^ = 37.5%), particularly in cases managed conservatively (OR 2.72, 95% CI 1.37–5.38, *p* = 0.004, I^2^ = 26.3%). Sensitivity analysis reinforced these findings, also demonstrating an association between ketorolac and hematomas requiring reoperation.

**Conclusion:**

Perioperative ketorolac is associated with an increased risk of hematoma following breast reduction surgery. While it provides effective opioid-sparing analgesia, its use should be carefully considered, especially in patients with higher bleeding risks. Further randomized trials are needed to refine safety recommendations.

**Level of Evidence II:**

This journal requires that authors assign a level of evidence to each article. For a full description of these Evidence-Based Medicine ratings, please refer to the Table of Contents or the online Instructions to Authors www.springer.com/00266

**Supplementary Information:**

The online version contains supplementary material available at 10.1007/s00266-025-05187-y.

## Introduction

Reduction mammaplasty is a widely performed surgical procedure aimed at alleviating symptoms associated with macromastia, including back pain, shoulder grooving, and skin irritation. Beyond functional benefits, the procedure also has aesthetic indications, improving breast contour and symmetry. According to the 2023 report from the American Society of Plastic Surgeons (ASPS), a total of 76,031 aesthetic breast reduction procedures were performed in the United States [1]. Despite its advantages, reduction mammaplasty is not without risks, with common complications including postoperative pain, wound healing disturbances, and bleeding. Among these, hematoma formation represents a particularly concerning postoperative event due to its potential need for further intervention [2–6].

Hematoma following breast reduction is a well-documented complication, with reported incidence rates varying from 1–9.3% [7–15]. The management of postoperative hematoma often necessitates additional procedures, ranging from aspiration under local anesthesia to surgical re-exploration [7, 16]. These interventions not only increase healthcare costs but also contribute to patient morbidity, including emergency consultations and hospital readmissions, thereby emphasizing the need for careful perioperative management.

In an effort to optimize postoperative recovery and patient comfort, multimodal analgesia strategies have been implemented, with ketorolac emerging as an effective non-opioid alternative for the management of moderate to severe pain. Studies have demonstrated that ketorolac significantly reduces the need for opioid analgesics, potentially by up to 50% [17–19]. This opioid-sparing effect is particularly advantageous given the gastrointestinal and respiratory complications associated with opioid use, including nausea, constipation, dependence, addiction, opioid use disorders, opioid-induced ventilatory impairment, and overdose-related deaths [17, 20]. Moreover, by mitigating the adverse effects linked to opioid consumption, ketorolac may represent a cost-effective strategy for postoperative pain control. Additionally, ketorolac has been noted to elicit a more rapid analgesic response compared to other nonsteroidal anti-inflammatory drugs (NSAIDs) [21–23].

Despite these benefits, the use of ketorolac in plastic surgery has been approached with caution due to concerns regarding its potential to increase postoperative bleeding risk. The drug's mechanism of action involves the inhibition of cyclooxygenase enzymes, which impairs platelet aggregation and prolongs bleeding time [24, 25]. Consequently, its perioperative use in reduction mammaplasty remains controversial, with concerns regarding an elevated risk of hematoma formation [21, 25, 26].

This issue continues to be debated in the literature, with no definitive consensus on the safety of ketorolac in the context of reduction mammaplasty. To address this gap, the present study represents the first systematic review and meta-analysis specifically evaluating whether ketorolac use is associated with an increased risk of hematoma following breast reduction.

## Methods

This systematic review and meta-analysis adhered to the methodologies outlined by the Cochrane collaboration and followed the PRISMA (Preferred Reporting Items for Systematic Reviews and Meta-Analyses) guidelines [27, 28].

### Eligibility Criteria

Study selection was guided by the PICOS framework (Population, Intervention, Comparison, Outcomes, and Study Design), with no restrictions on language (Table 1).

**Table 1:** PICOS framework. Eligibility criteria according to PICOS

Parameter	Inclusion criteria	Exclusion criteria
Population	Patients who underwent breast reduction surgery	Patients who underwent other surgical procedures
Intervention	Use of ketorolac perioperatively	Inability to determine the number of patients who received perioperative ketorolac
Comparison	No ketorolac use or use of an alternative analgesic	–
Outcomes	Postoperative hematoma	Studies not reporting hematoma as an outcome
Study design	Observational studies (case-control, cohort, retrospective reviews), randomized controlled trials	Conference abstracts, case reports and series, narrative or systematic reviews, editorials, letters

### Information Sources and Data Collection

A comprehensive literature search was conducted in Medline Plus, Embase, Cochrane Central, and Web of Science to identify studies published up to March 1, 2025 that investigated the association between perioperative ketorolac use and hematoma incidence following breast reduction surgery. The search strategy incorporated a combination of keywords, including "ketorolac," "toradol," "reduction mammaplasty," and "breast reduction," as outlined in Supplementary Table 1.

Studies screening and data extraction were performed independently by two researchers (V.F. and G.D.) following predefined eligibility. Any discrepancies between reviewers were addressed through discussion with a third investigator (E.F.R.D.).

The data extracted from each study were categorized into three main groups. First, identification details included the study author, country of origin, and study period. Second, sample characteristics were collected, such as the total number of patients in each study, the proportion of individuals who received ketorolac versus those who did not, and demographic data like age and body mass index. Third, intervention and outcome data were examined, covering administered ketorolac doses, timing of administration (intraoperative or postoperative), and the method used to assess outcomes. Additionally, hematoma incidence was documented, distinguishing between cases that required surgical intervention and those managed conservatively.

### Endpoints

The primary endpoint was hematoma formation, defined as postoperative internal bleeding or extensive subcutaneous hematoma requiring intervention. Secondary endpoints included the classification of hematomas based on the type of intervention required, distinguishing between non-operative management—such as local drainage, aspiration, or simple clinical observation—and surgical intervention performed in the operating room, including operative evacuation or re-exploration. Due to variations in hematoma definitions across studies, detailed descriptions are provided in Supplementary Table 2.

### Quality and Bias Assessment

The quality assessment and risk of bias evaluation were conducted using the Quality In prognosis studies (QUIPS) tool for observational studies and Revised Cochrane risk-of-bias tool for randomized trials (RoB 2) for randomized controlled trials, in accordance with the recommendations of the Cochrane prognosis methods group [29].

QUIPS tool examines six key domains: study participation, attrition, measurement of prognostic factors, outcome assessment, confounding control, and statistical analysis with reporting. Each domain was classified as having a low, moderate, or high risk of bias [30]. Studies were categorized as low risk if they had no more than one moderate-risk domain, high risk if they contained at least one high-risk domain or three or more moderate-risk domains, and moderate risk if they did not meet either criterion [31].

The RoB 2 tool assesses bias in randomized trials across five domains: randomization process, deviations from intended interventions, missing outcome data, outcome measurement, and selective reporting. Each domain is rated as low risk, some concerns, or high risk, and the overall risk of bias is determined by the highest concern across domains. A study is rated as low risk if all domains are low risk, some concerns if at least one domain raises concerns, and high risk if multiple domains raise concerns or at least one is high risk [32].

Two independent reviewers (V.F. and G.D.) conducted the evaluations. Any discrepancies were resolved by consulting a third reviewer (E.F.R.D.). To assess publication bias, funnel plots were applied to each outcome. Egger's test was not performed due to the limited number of studies included in the analysis.

### Statistical Analysis

The statistical analysis was conducted using the"meta"package in R version 4.4.2 (The R Foundation, 2023). To compare the incidence of binary outcomes between treatment groups, odds ratios (OR) with 95% confidence intervals (CI) were computed. Heterogeneity was evaluated using Cochran’s Q test and the I^2^ statistic, with low heterogeneity defined as *p* > 0.10 and I^2^ < 25%. A random-effects model was used for all outcomes, with the Mantel–Haenszel method for statistical analysis and the DerSimonian and Laird approach for heterogeneity estimation. Sensitivity analyses were performed using a leave-one-out approach to assess the impact of individual studies on the overall pooled estimates. Additionally, studies identified as having a high risk of bias were excluded to evaluate their influence on the results. Statistical significance was established at *p* < 0.05.

## Results

**Fig. 1:**
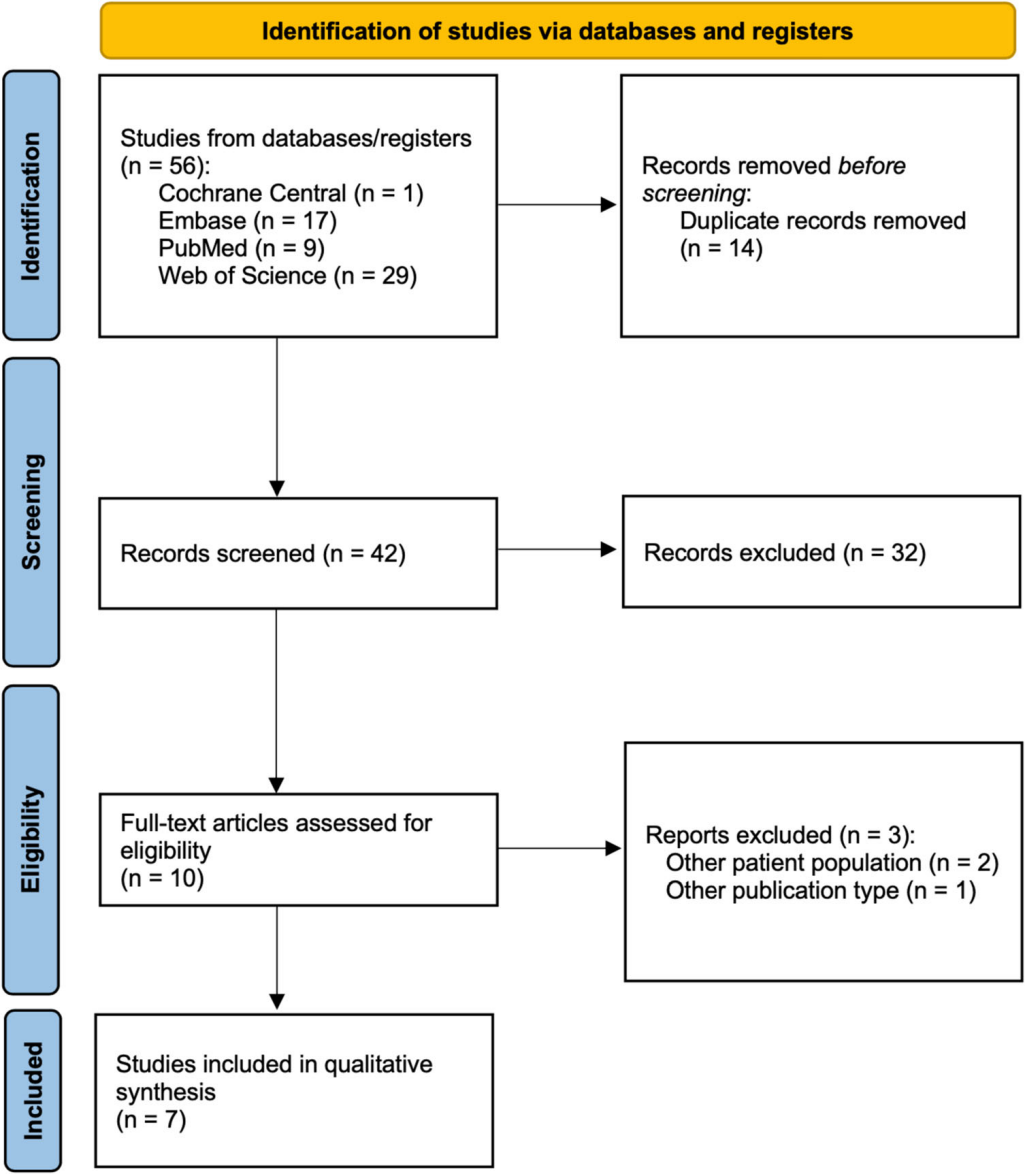
PRISMA flowchart. PRISMA flow diagram of study screening and selection

A systematic database search yielded a total of 56 records. Following the removal of duplicates and an initial screening of titles and abstracts, ten studies underwent a full-text review. Two articles were excluded due to involving a different study population, and one was removed as it was a conference abstract. Ultimately, seven studies fulfilled the eligibility criteria for the systematic review. Of these, six were observational studies and one was a randomized controlled trial (Fig. 1). The main characteristics of the included studies are summarized in Table 2.

**Table 2:** Baseline patient and study characteristics. Summary of the main characteristics of the included studies

Study	Country	Study design	Period	Target comparison	Target sample, No.	Study sample sex	Age, ± SD, range (−)	BMI, ± SD, range (−)	Ketorolac use (No.)	Ketorolac dosage (± SD, IQR, No.)	Outcome assessment
Barkho et al. [20]	Canada	Retrospective case-control	2002–2016	Hematoma requiring operation versus no hematoma (ketorolac vs. no ketorolac)	40 versus 40 (31 vs. 49)	NA	44.4 ± 13.3 versus 44.5 ± 13.2	28.5 ± 5.1 versus 30.0 ± 5.1	IO (n = 25), PO (n = 6)	IV, means 27.0 mg (± 6.2) versus 27.3 mg (± 6.1)	Cases and controls identified through the coding system of hospitals’ electronic medical records
Blomqvist et al. [16]	Sweden	Retrospective review	1990–1991	Perioperative ketorolac versus no perioperative ketorolac	5 versus 288	Female	35.1 (16–75)	23.7 (18.1-44.8)	IO, PO	IM, 30 mg	Medical records
Cawthorn et al. [25]	Canada	Retrospective review	2004–2007	Perioperative ketorolac versus no perioperative ketorolac	127 versus 252	Female	42.3 ± 12.9 versus 40.0 ± 13.0	31.6 ± 6.2 versus 31.6 ± 5.9	IO (n = 124), up to 2 h PO (n = 3)	IV, 15 mg (n = 119) or 30 mg (n = 8)	Surgeon’s postoperative notes or follow-up reports
Choi et al. [33]	USA	Randomized controlled trial	2020–2023	Perioperative ketorolac versus placebo	24 versus 10	Female	NA	NA	Immediate PO (n = 24)	IV, 15 mg (n = 12) or 30 mg (n = 12)	Immediate postoperative and on follow-up at 1 week
Firriolo et al. [34]	USA	Retrospective review	2007–2017	Perioperative ketorolac versus no perioperative ketorolac	389 versus 111	Female	18.1 ± 2.3 versus 18.0 ± 1.8	NA	IO (n = 356), PO (n = 170), both (n = 137)	IV, IO: median 30 mg (0 mg); PO: median 90 mg (30 mg)	Clinical notes, operative reports, anesthesia records
Nguyen et al. [10]	USA	Retrospective review	2012–2014	PO ketorolac versus no PO ketorolac	75 versus 303	NA	NA	NA	PO (up to 5 days)	IV, 15 mg or 30 mg (from 1 to 11 doses)	Follow-up clinical documentation
Stanek et al. [7]	USA	Retrospective review	2012–2022	Hematoma versus no hematoma (ketorolac vs. no ketorolac)	53 versus 1,701 (1,340 vs. 414)	Female	18.64 ± 3.08 versus 17.84 ± 2.40	28.2 ± 5.5 versus 29.1 ± 6.8	IO, PO	NA	Clinical notes, operative and anesthesia reports, and medication charts

*BMI* Body mass index, *IQR* Interquartile range, *IO* Intraoperative, *IM* Intramuscular, *IV* Intravenous, *PO* Postoperative, *SD* Standard deviation, *NA* Not available

**Fig. 2:**
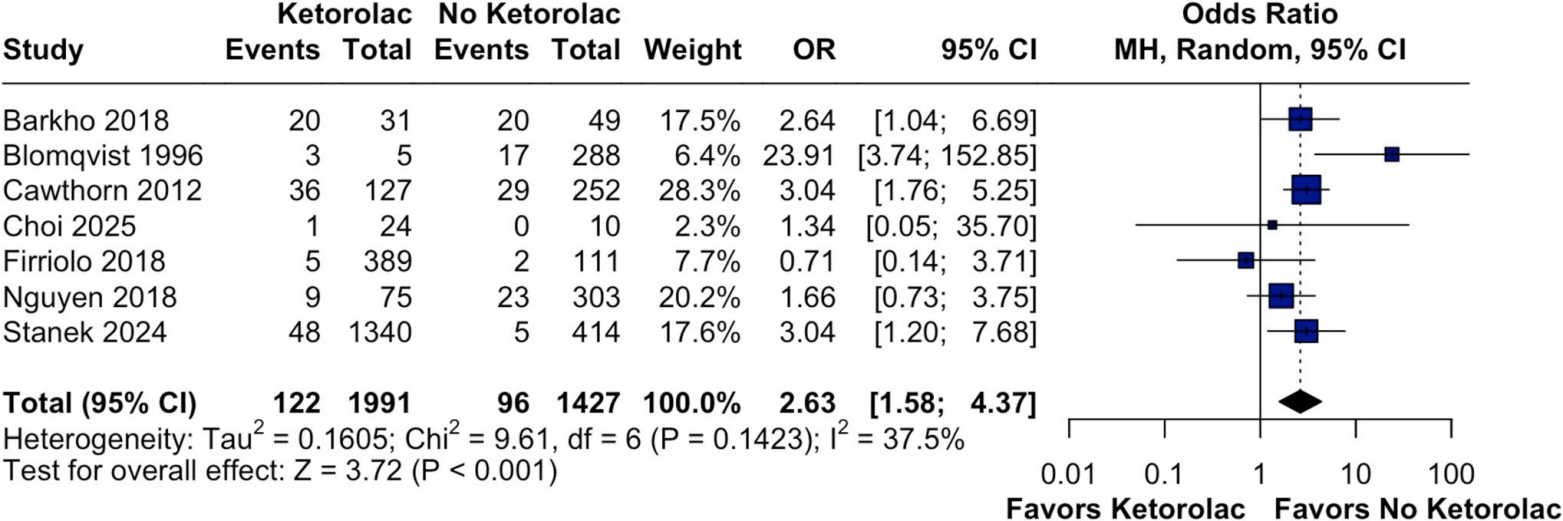
Forest plot illustrating the assessment of hematoma outcomes. The analysis indicates that the ketorolac group exhibited an increased risk of developing any type of hematoma

A total of 3418 patients were included in the analysis, with 1991 receiving perioperative ketorolac and 1427 not receiving ketorolac. The overall incidence of hematoma was 122 events (6.13%) in the ketorolac group and 96 events (6.73%) in the no-ketorolac group. The pooled OR for hematoma formation associated with ketorolac use was 2.63 (95% CI 1.58–4.37, *p* < 0.001, I^2^ = 37.5%), indicating a significantly higher risk of hematoma in the ketorolac group (Fig. 2).

### Subgroup Analysis

#### Hematomas Not Requiring Reoperation

**Fig. 3:**
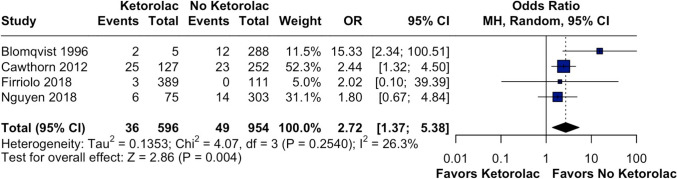
Forest plot of hematomas not requiring reoperation. The analysis shows a higher risk of hematomas not requiring reoperation in the ketorolac group

In this subgroup, which included 1550 patients (596 in the ketorolac group and 954 in the no-ketorolac group), the overall incidence of hematomas managed conservatively was 36 events (6.04%) in the ketorolac group and 49 events (5.13%) in the no-ketorolac group. The pooled OR was 2.72 (95% CI 1.37–5.38, *p* = 0.004, I^2^ = 26.3%), suggesting a significantly increased risk of hematoma requiring minor intervention in patients receiving ketorolac (Fig. 3).

#### Hematomas Requiring Reoperation

**Fig. 4:**
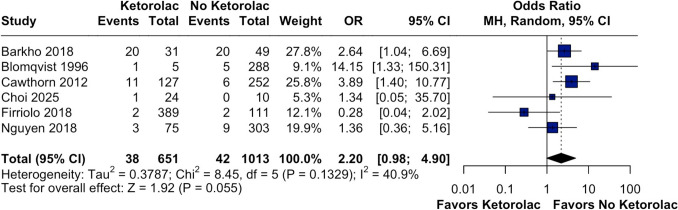
Forest plot of hematomas requiring reoperation. The meta-analysis suggested a potential increase in risk in the ketorolac group, though without statistical significance

In contrast, the association between ketorolac use and hematomas requiring surgical reoperation was not statistically significant. This subgroup included 1664 patients (651 in the ketorolac group and 1013 in the no-ketorolac group), with a total of 38 events (5.84%) in the ketorolac group and 42 events (4.15%) in the no-ketorolac group. The pooled OR was 2.20 (95% CI 0.98–4.90, *p* = 0.055, I^2^ = 40.9%), indicating a potential increase in risk, but without reaching statistical significance (Fig. 4).

### Quality and Bias Assessment

The risk of bias assessment using the QUIPS tool indicates that most studies had a low risk of bias, ensuring their reliability in the systematic review. However, concerns were noted in two key domains: participation and confounding. Regarding the participation domain, three studies presented moderate concerns [7, 10, 16], while five studies showed confounding concerns [7, 10, 16, 20, 34]. Overall, three studies were classified as having a low risk of bias [20, 25, 34]. Nguyen et al. and Stanek et al. [7, 10] were classified as having moderate risk due to concerns in both the participation and confounding domains. Notably, Blomqvist et al. [16] exhibited high concerns in the confounding domain, leading to their classification as high-risk study. A detailed summary of the risk of bias assessment using the QUIPS tool is provided in Supplementary Fig. 1. The quality and risk of bias assessment of Choi et al. [33] using the RoB 2 tool indicated a low risk of bias across all domains, resulting in an overall low-risk classification.

The funnel plot for overall hematoma incidence and hematomas not requiring reoperation (Supplementary Figs. 2 and 3) demonstrated slight asymmetry, suggesting potential publication bias or heterogeneity among included studies. In contrast, the funnel plot for hematomas requiring reoperation (Supplementary Fig. 4) appeared more symmetrical, suggesting a lower likelihood of publication bias in this subset of data.

### Sensitivity Analysis

A sensitivity analysis was conducted for hematoma outcomes by removing Blomqvist et [16], which eliminated heterogeneity (I^2^ = 0%) and yielded a similar adjusted OR of 2.44 (95% CI 1.70–3.50, *p* < 0.0001), reinforcing the consistency of the findings (Supplementary Fig. 5). Regarding hematomas not requiring reoperation, leave-one-out analysis showed that excluding Blomqvist et al. [16] reduced heterogeneity from 26% to zero. This adjustment resulted in similar findings (OR = 2.23, 95% CI 1.34–3.73, *p* = 0.002), further supporting the robustness of the results (Supplementary Fig. 6). In the case of hematomas requiring reoperation, a leave-one-out sensitivity analysis excluding Firriolo et al. (2018) led to a reduction in heterogeneity from 52% to zero. This adjustment provided statistically significant evidence of an increased risk of hematomas requiring reoperation in the ketorolac group (OR = 2.86, 95% CI 1.60–5.12, *p* = 0.0004) (Supplementary Fig. 7). The sensitivity analysis based on the risk of bias assessment yielded similar results, as Blomqvist et al. [16] was the only study identified with a high risk of bias.

## Discussion

To the best of our knowledge this is the first systematic review and meta-analysis evaluating the association between perioperative ketorolac administration and hematoma risk following specifically breast reduction. We analyzed data from six observational studies comprising a total of 3384 patients, of whom 1967 received ketorolac and 1417 did not. Our findings indicate a significant association between perioperative ketorolac administration and hematoma formation, particularly in cases managed conservatively. Sensitivity analysis also demonstrated a significant association between ketorolac use and hematomas requiring reoperation.

Our quantitative synthesis found a lower absolute incidence of hematoma in the ketorolac group (n = 121/1967, 6.15%) than in the control group (n = 96/1417, 6.78%), which may seem counterintuitive. However, the association identified in the meta-analysis is driven by the odds ratio, which accounts for the relative group sizes and weighting of individual studies, rather than by raw incidence alone. Except for Firriolo et al. [34], all primary studies individually showed either a significant or non-significant trend toward an association between ketorolac and hematomas. Once synthesized, this association became strong and was later confirmed by sensitivity analysis.

Ketorolac tromethamine, a widely used NSAID, is recognized for its opioid-sparing effects, yet its perioperative use remains debated due to potential bleeding risks. As a competitive and nonselective COX inhibitor, ketorolac reduces thromboxane A2 production, impairing platelet aggregation. Although it transiently prolongs bleeding time in healthy individuals, it does not substantially alter primary coagulation or fibrinolytic pathways. However, surgical stress may influence platelet function, potentially modifying ketorolac’s effects [36–38].

Reports of increased bleeding with ketorolac date back to Garcha [39], who documented four cases of postoperative breast surgery hematomas within ten days, raising concerns about a possible link to postoperative ketorolac use. Subsequent studies, including Strom et al. [40], found that while overall associations between ketorolac use and both gastrointestinal and surgical site bleeding were minimal, the risk became clinically relevant in patients receiving higher doses, in older populations, and with prolonged administration beyond 5 days.

Despite these concerns, evidence on the bleeding risk associated with ketorolac remains controversial. Stephens et al. [21] conducted a meta-analysis incorporating six studies (three randomized clinical trials, two retrospective reviews, and one case series) and found no significant association between ketorolac and hematoma formation across various plastic surgery procedures, including breast reduction and breast augmentation. Similarly, Gobble et al. [26] performed a meta-analysis of randomized clinical trials covering various surgical procedures—including upper abdominal surgery, hysterectomy, cesarean section, tonsillectomy, and arthroplasty—and found no significant increase in perioperative bleeding related to ketorolac use.

Conversely, some studies have identified an increased bleeding risk with ketorolac use in other surgical procedures, such as appendectomy [41] and tonsillectomy [42, 43]. These studies analyzed more narrowly defined surgical procedures and patient populations, yielding stronger associations between ketorolac and bleeding complications. Similarly, our study adopts a more focused approach, assessing a proportionally larger sample within a defined PICOS framework, leading to more reliable findings.

This discrepancy likely reflects differences in sample sizes. Blomqvist et al. [16] had the smallest ketorolac-exposed group but a control population 57 times larger, whereas Stanek et al. [7] had the largest ketorolac-exposed group, three times bigger than its control. Such variations introduce biases, particularly in population selection and confounding factors, as highlighted in the risk-of-bias analysis.

Barkho et al. [20] was the only study in our review to use a case-control design, a reasonable approach given the infrequent occurrence of hematoma events. This design also mitigates confounding factors by matching patients based on baseline characteristics. However, to align with our quantitative synthesis, we restructured the data, defining groups by drug exposure rather than event occurrence. This adjustment reintroduced potential confounders that the original design minimized. Moreover, this methodological difference may explain why Barkho et al. [20], in our analysis, showed a statistically significant association between ketorolac and hematoma occurrence—something not observed in the original study, despite a strong trend toward association.

Although not directly analyzed in this meta-analysis due to limited primary data, key aspects of perioperative ketorolac and NSAID use include pain control and opioid reduction. These factors were assessed in the only randomized controlled trial in our review. Choi et al. found a statistically significant improvement in pain resolution with both 15 and 30 mg of ketorolac compared to placebo. While opioid use in the postoperative anesthesia care unit showed no significant between-group difference, post-discharge opioid consumption was lower in patients receiving perioperative ketorolac, particularly at 30 mg [34]. Firriolo et al. supports these findings, associating ketorolac use with reduced intraoperative and postoperative opioid consumption, consistent with prior research [17–19, 33]. Given the ongoing opioid crisis, intravenous ketorolac remains a valuable adjunct in multimodal analgesia to minimize opioid-related adverse effects such as nausea, vomiting, respiratory depression, and prolonged hospital stays [44, 45, 46]. However, its use should be carefully weighed against the potential increased hematoma risk in reduction mammaplasty patients.

Several limitations must be acknowledged. This meta-analysis is based on retrospective observational studies, which lack the rigor of randomized controlled trials and are prone to selection bias, incomplete documentation, and residual confounding. Furthermore, as discussed, study heterogeneity poses a challenge due to variations in sample size, surgical techniques, ketorolac dosing regimens, and hematoma definitions. While a sensitivity analysis was conducted to mitigate these issues, inconsistencies in hematoma classification necessitated a subgroup analysis differentiating hematomas requiring surgical intervention from those managed conservatively.

Additionally, we were unable to assess confounding factors such as hypertension, anticoagulant use, obesity, and other comorbidities that may contribute to hematoma risk, as well as operative variables including intraoperative blood pressure control, surgical technique, and drain use. For example, Cawthorn et al. [25] reported a higher prevalence of hypertension in the ketorolac group, which may have confounded their results. Due to insufficient studies reporting uniform baseline characteristics, a meta-regression analysis could not be performed to adjust for these variables. Another aspect that remains to be clarified is the hematoma risk of the ketorolac administration at different perioperative and postoperative time points. This is a significant gap in the literature, as no studies comparing different timing strategies for ketorolac use in breast reduction were available at the time of our systematic review.

Similarly, due to limited data availability, we were unable to perform a pooled analysis to evaluate whether the association with hematoma formation differs between between 15 and 30 mg doses. On future studies, it would be important to explore the impact of the different dosages of ketorolac.

## Conclusion

This study highlights the potential association between perioperative ketorolac use and an increased risk of hematoma following reduction mammaplasty, emphasizing the need for cautious use in this surgical setting. While ketorolac provides significant opioid-sparing benefits, its impact on bleeding risk cannot be overlooked in clinical practice, and warrants careful patient selection and individualized risk assessment, especially in patients with additional bleeding risk factors. Further high-quality, randomized controlled studies are necessary to clarify its safety profile and optimize perioperative analgesic strategies in plastic surgery.

## Supplementary Information

Below is the link to the electronic supplementary material.

Supplementary file1 (DOCX 1169 kb)

## Data Availability

All data generated or analyzed during this review are available to the corresponding author upon request.

## Abbreviations

CI: Confidence interval
NSAID: Nonsteroidal anti-inflammatory drug
OR: Odds ratio
QUIPS: Quality In prognosis studies
