# Supplementary material for: Perioperative Ketorolac and Hematoma Following Breast Reduction: A Systematic Review and Meta-analysis
Source: Aesthetic Plast Surg. 2025 Sep 19;50(3):1057–66. doi: 10.1007/s00266-025-05187-y (PMC12992376; doi:10.1007/s00266-025-05187-y)

**SUPPLEMENTAL APPENDIX**

**Supplementary Table 1.** *Search strategy*. Search strategy by database.

| **Database** | **Search strategy** |
| --- | --- |
| **Cochrane Central** | #1: MeSH descriptor: [Ketorolac] explode all trees  #2: MeSH descriptor: [Tromethamine] explode all trees #3: MeSH descriptor: [Ketorolac Tromethamine] explode all trees (#1 OR #2 OR #3) AND ("reduction mammoplasty" OR "reduction mammaplasty" OR "reduction mammaplasties" OR "reduction mammoplasties" OR "breast reduction" OR "mammoplasty, reduction" OR "mammoplasties, reduction" OR "mammaplasty, reduction" OR "mammaplasties, reduction" OR "reductive breast correction") |
| **Embase** | (‘ketorolac'/exp OR 'ketorolac trometamol'/exp OR 'trometamol'/exp) AND (reduction mamm*plast* OR 'breast reduction'/exp) |
| **PubMed** | (ketorolac OR "ketorolac" [Mesh] OR toradol OR tromethamine OR ketorolac trometamol) AND ("reduction mamm*plast*" OR "breast reduction" OR "mamm*plast*, reduction" OR reductive breast correction) |
| **Web of Science** | (ALL=(ketorolac) OR ALL=(toradol) OR ALL=(tromethamine) OR ALL=(ketorolac trometamol)) AND (ALL=(reduction mamm*plast*) OR ALL=(breast reduction) OR ALL=(mamm*plast*, reduction) OR ALL=(reductive breast correction)) |

**Supplementary Table 2.** *Hematoma definitions*. Definition of hematoma across studies.

| **Study** | **Definition of hematoma** |
| --- | --- |
| Barkho et al. (2018)^20^ | Cases and controls were identified through the coding system utilized by our hospitals’ electronic medical records. Coders searched for “hemorrhage,” “hematoma”. |
| Blomqvist et al. (1996)^16^ | Postoperative bleeding requiring reoperation or widespread subcutaneous hematoma in or around the breast, not suitable for reoperation but in some cases requiring drainage. |
| Cawthorn et al. (2012)^26^ | Primary outcome: hematoma formation requiring surgical evacuation, was defined as patients who developed hematomas during the first 24 hr postoperatively, which required return to the operating room for surgical re-exploration and evacuation.  Secondary outcome: hematoma formation not requiring surgical re-exploration. |
| Choi et al. (2025)^33^ | Delayed hematoma, which was evacuated surgically. |
| Firriolo et al. (2018)^34^ | Hematoma was defined as a clinically identifiable soft tissue swelling secondary to a localized collection of blood that required intervention, whether that be drainage under local anesthesia or operative evacuation. |
| Nguyen et al. (2018)^10^ | Primary outcome: operative hematoma formation requiring surgical evacuation  Secondary outcome: nonoperative hematoma formation (surgical evacuation was not required) |
| Stanek et al. (2024)^7^ | Postoperative hematomas of any kind, whether or not an intervention was required. Hematomas were defined as localized collections of blood causing clinically identifiable swelling or palpable firmness. |

**Supplemental Figure 1.** Quality and bias assessment (QUIPS tool)

**
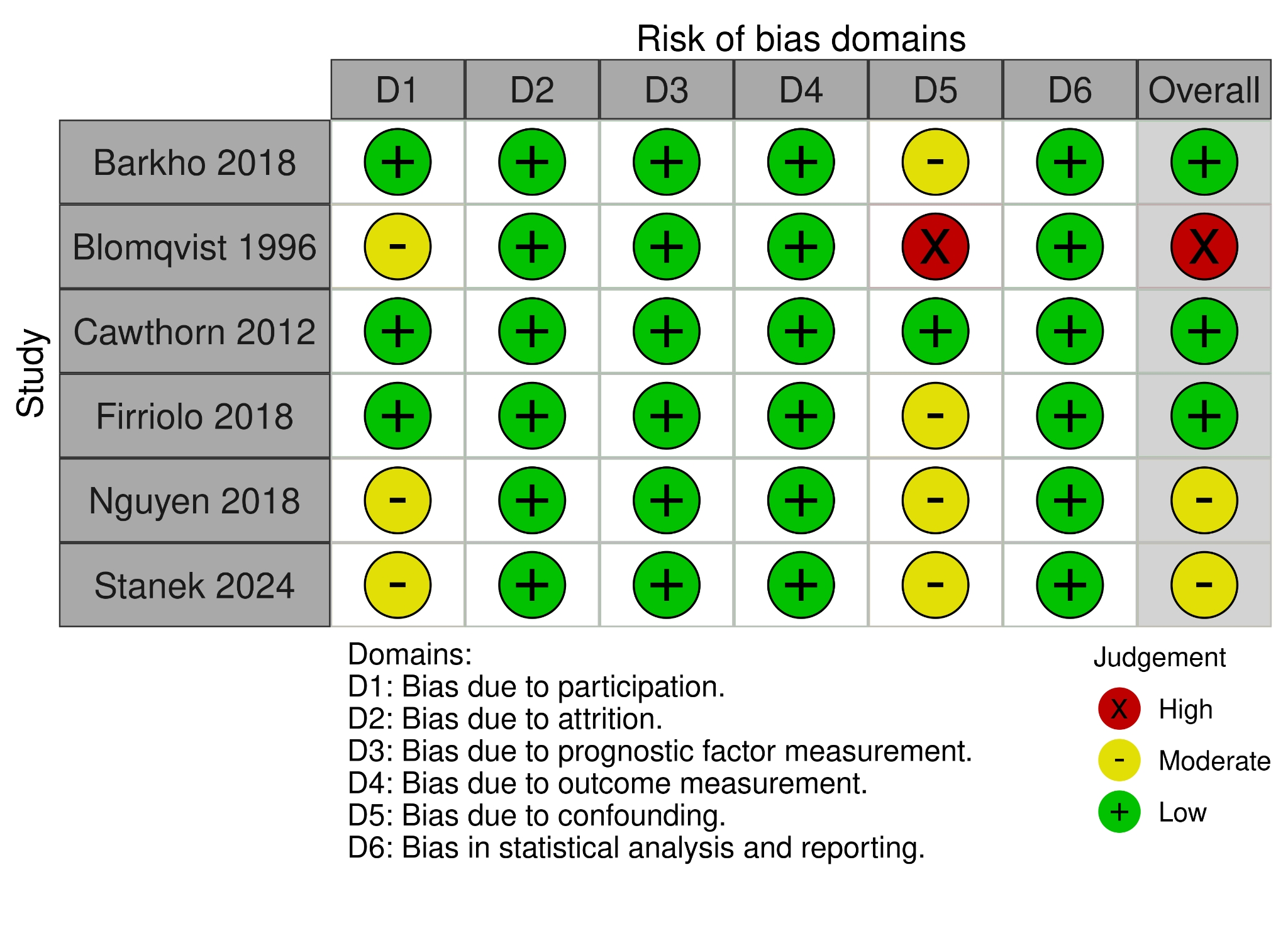

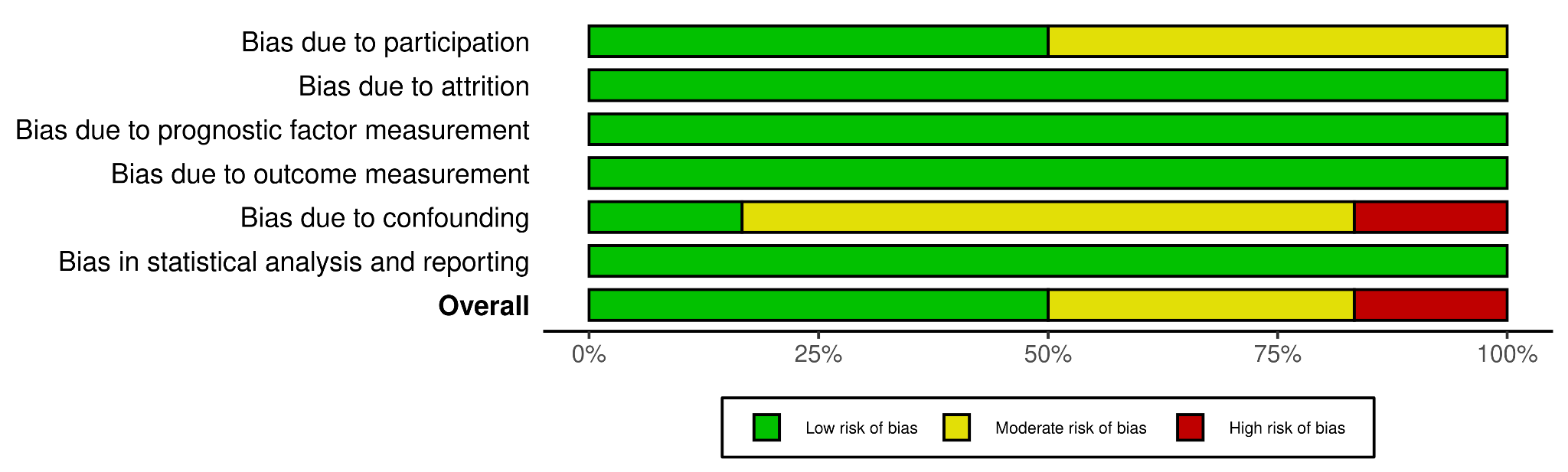
**

**Supplemental Figure 2.** *Funnel plot of hematoma outcomes*.


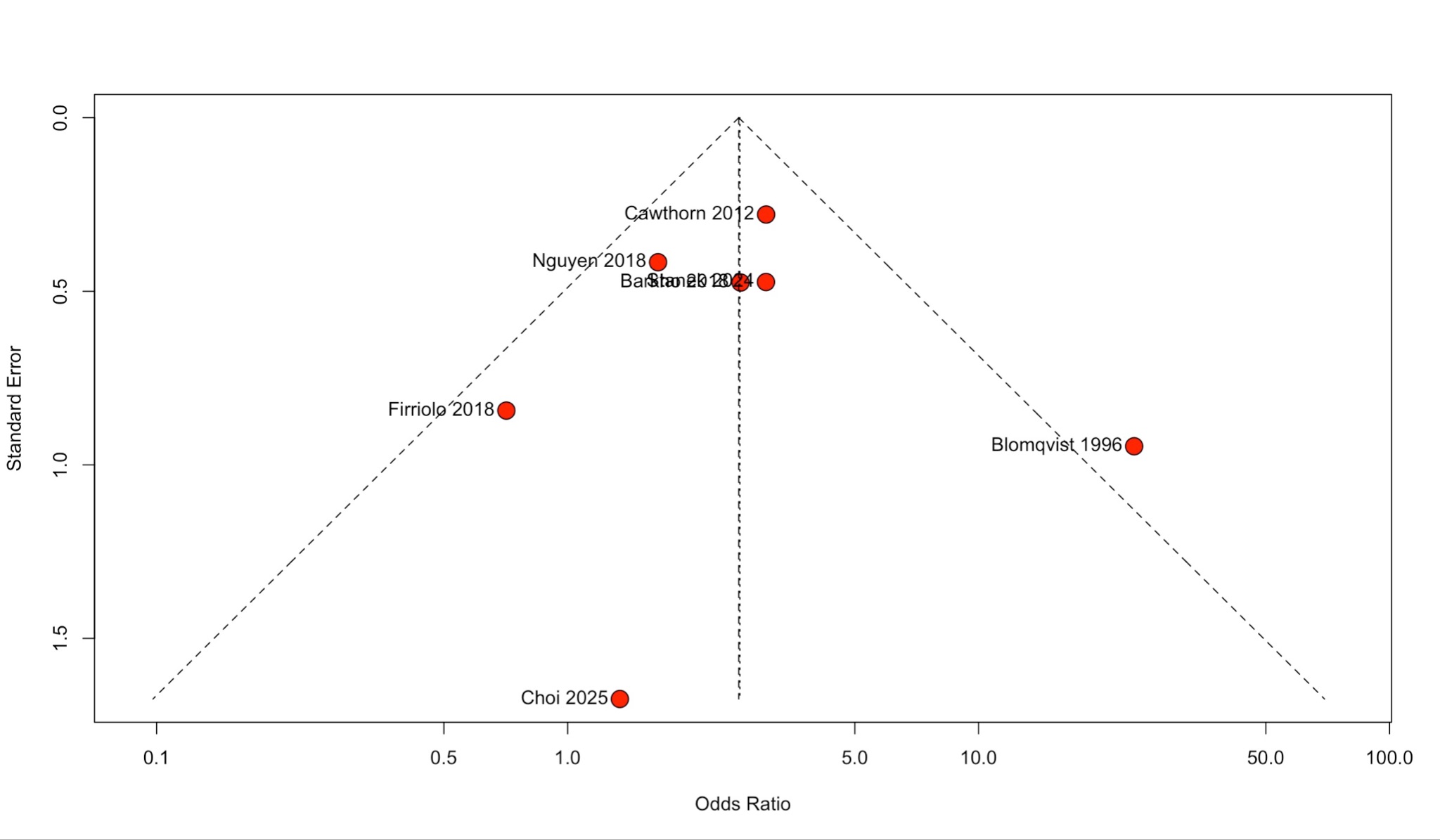


**Supplemental Figure 3.** *Funnel plot of hematomas not requiring reoperation*.


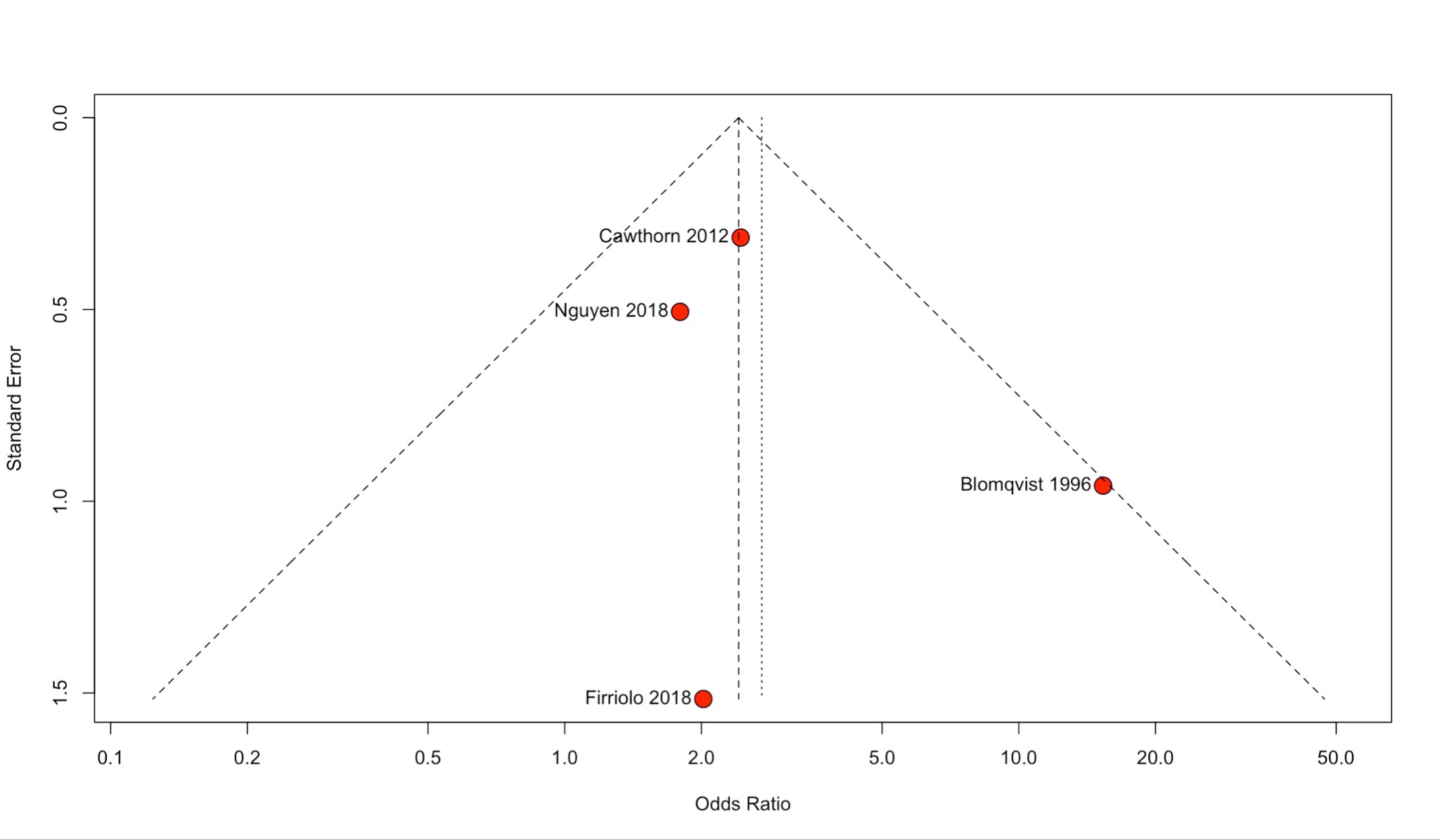


**Supplemental Figure 4.** *Funnel plot of hematomas requiring reoperation*.


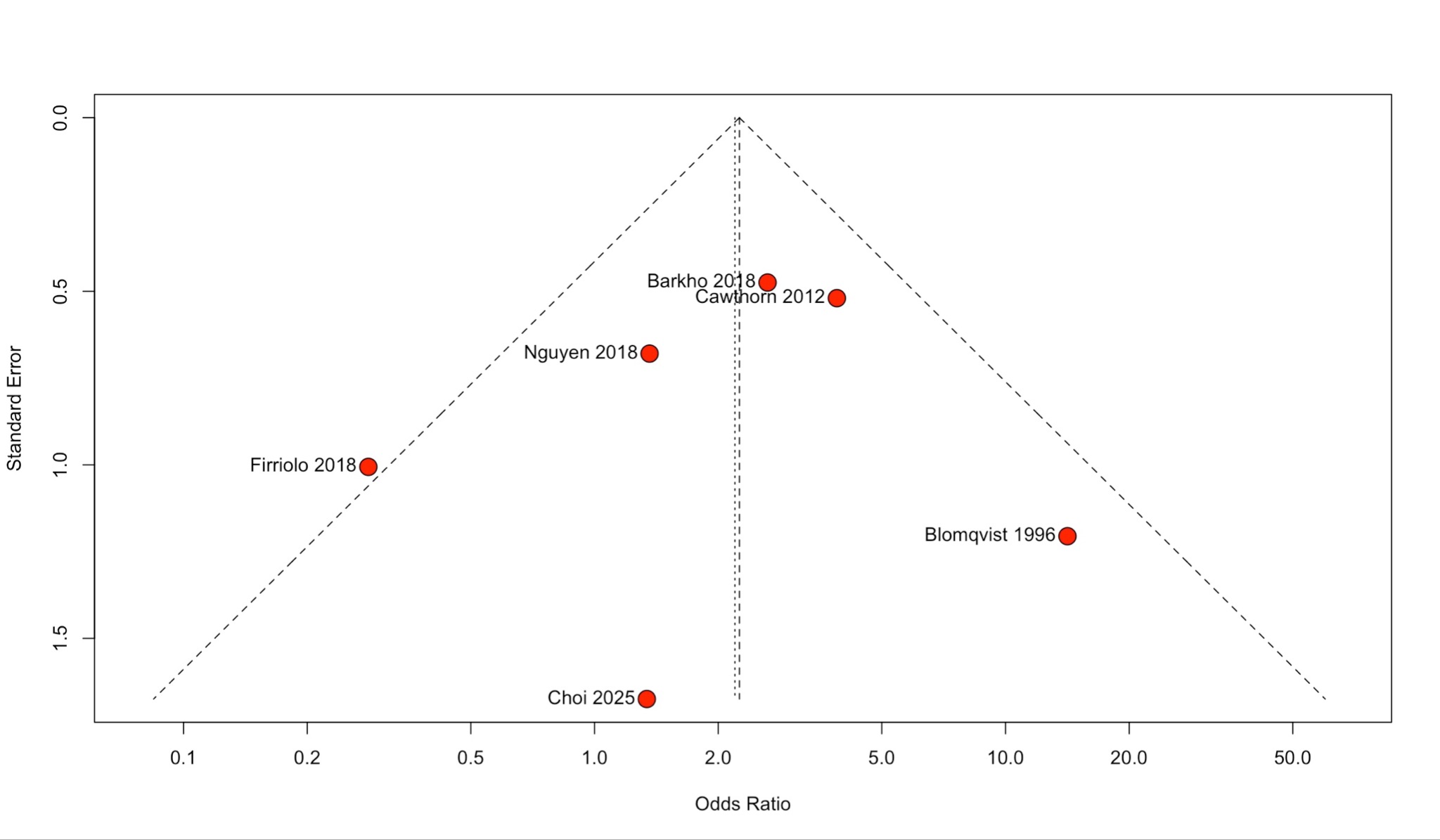


**Supplemental Figure 5.** *Forest plot of hematoma outcomes after leave-one-out analysis*. Excluding Blomqvist et al. (1996) removed heterogeneity, confirming the increased hematoma risk in the ketorolac group.

**
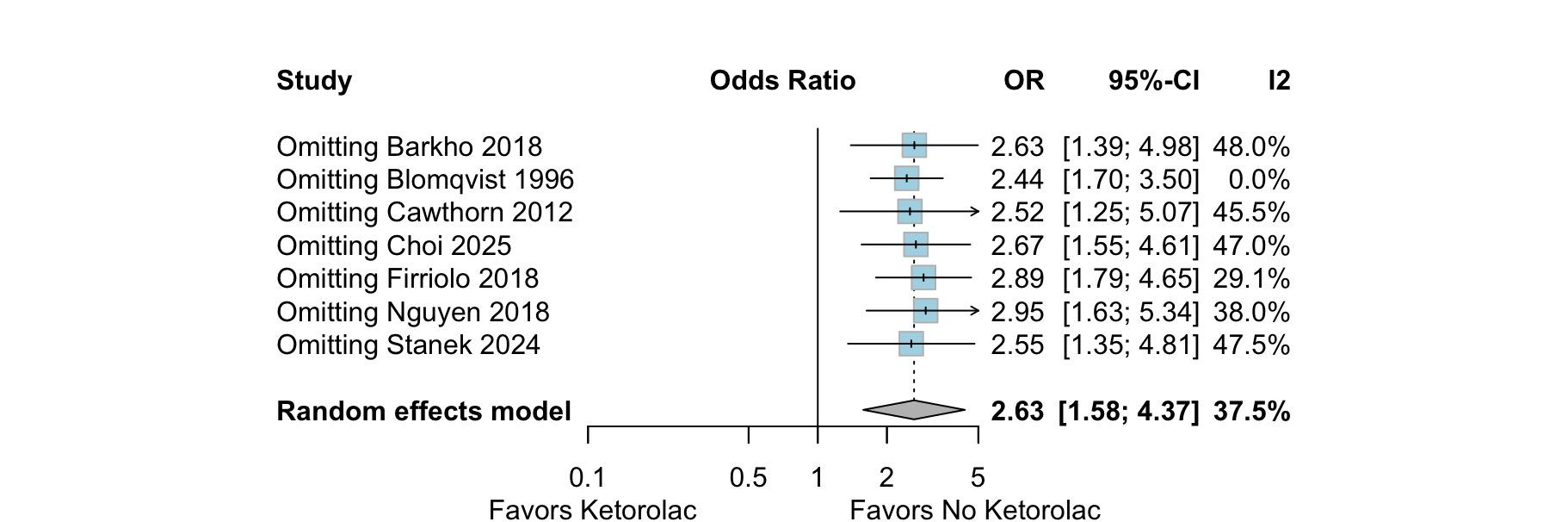
**

**Supplemental Figure 6.** *Forest plot of hematomas not requiring reoperation*. The exclusion of Blomqvist et al. (1996) eliminated heterogeneity, reaffirming the increased risk of hematomas not requiring reoperation in the ketorolac group.


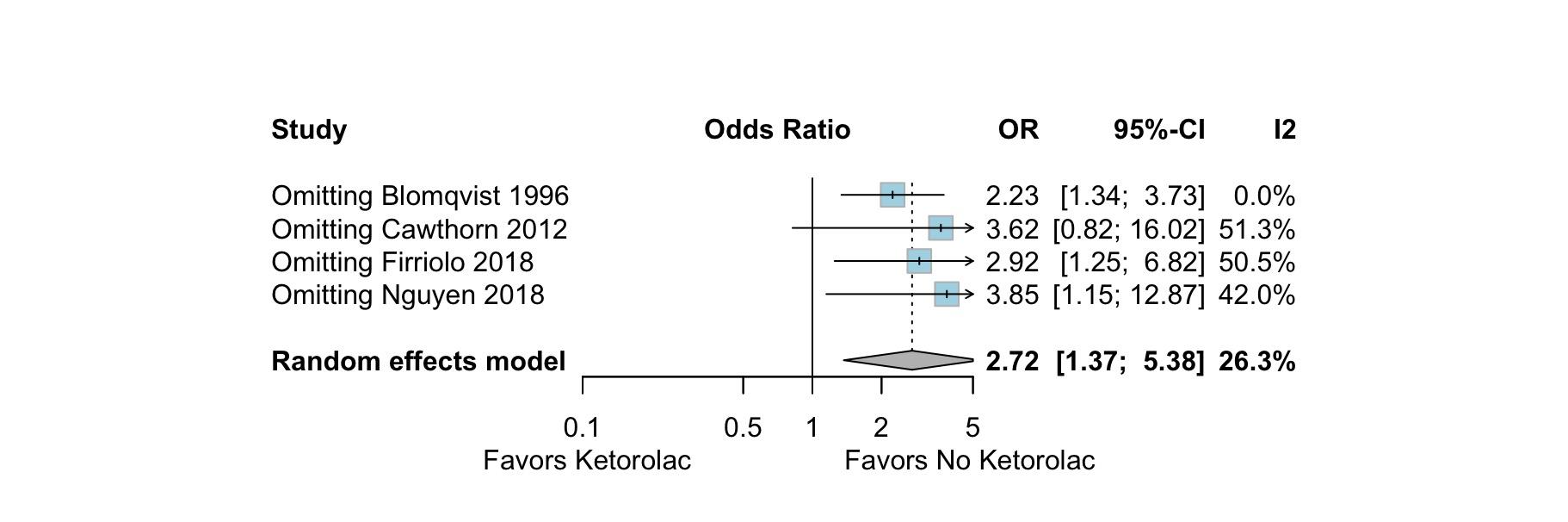


**Supplemental Figure 7.** *Forest plot of hematomas requiring reoperation*. The exclusion of Firriolo et al. (2018) reduced heterogeneity from 52% to zero, which provided statistically significant evidence of an increased risk of hematomas requiring reoperation in the ketorolac group.


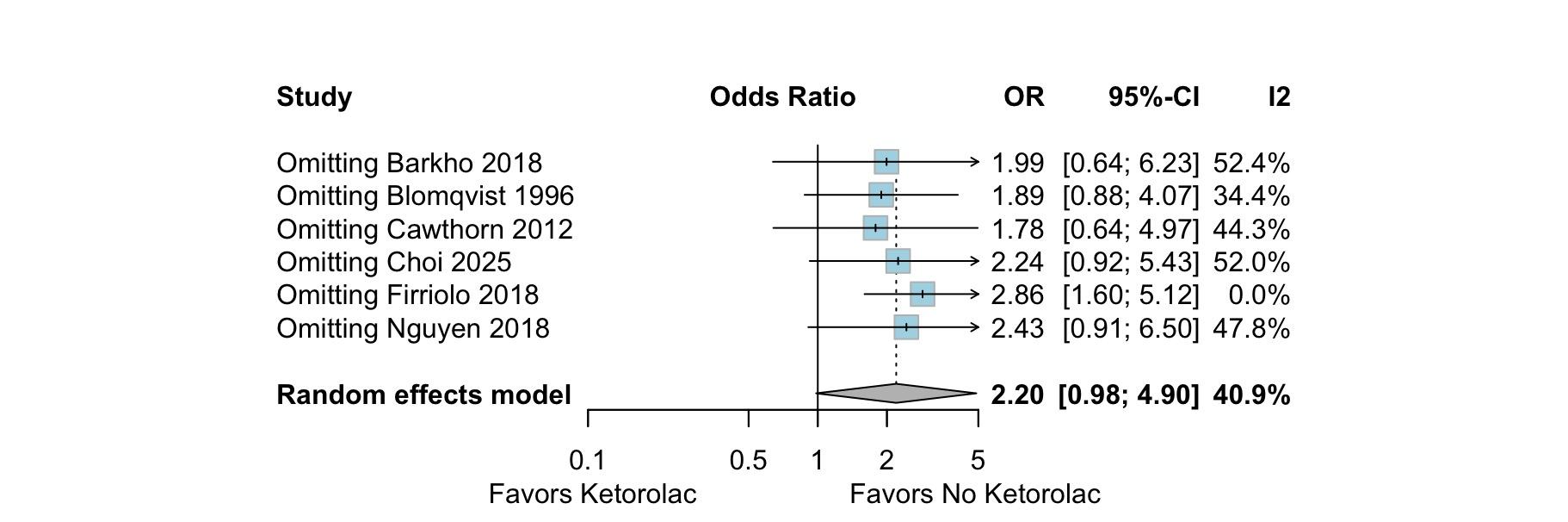

Supplement: Supplementary file 1 — Supplementary file1 (DOCX 1169 kb) [file 266_2025_5187_MOESM1_ESM.docx]
